# Supplementary material for: Higher Sodium Intake Assessed by 24 Hour Urinary Sodium Excretion Is Associated with Non-Alcoholic Fatty Liver Disease: The PREVEND Cohort Study
Source: J Clin Med. 2019 Dec 6;8(12):2157. doi: 10.3390/jcm8122157 (PMC6947413; doi:10.3390/jcm8122157)
Supplement: Supplementary file 1 [file jcm-08-02157-s001.pdf]

Supplemental Data

Table S1. Multivariable regression analysis demonstrating the positive association of an elevated Fatty Liver Index (FLI ≥ 60) with averaged 24-h sodium excretion (two collections) after adjustment for clinical and laboratory covariates in 3,100 women and in 3,032 men separately.

	**Model 1**		**Model 2**		**Model 3**		**Model 4**	
**Women**								
	OR (95%CI)	*P* value	OR (95%CI)	*P* value	OR (95%CI)	*P* value	OR (95%CI)	*P* value
**Age** (years)	1.06 (1.05-1.07)	< 0.001	1.03 (1.03-1.04)	< 0.001	1.01 (1.00-1.02)	0.043	1.00 (0.98-1.01)	0.810
**Sodium excretion per 24-h** (1 SD increment)	1.70 (1.53-1.90)	< 0.001	1.67 (1.48-1.87)	< 0.001	1.69 (1.50-1.91)	< 0.001	1.47 (1.28-1.69)	< 0.001
**Type 2 diabetes mellitus** (yes/no)			5.27 (3.65-7.60)	< 0.001	5.50 (3.12-9.70)	< 0.001	0.64 (0.32-1.27)	0.199
**History of cardiovascular disease** (yes/no)			1.85 (1.19-2.90)	0.007	1.34 (0.81-2.22)	0.249	1.03 (0.56-1.90)	0.918
**Hypertension** (yes/no)			2.61 (2.10-3.23)	< 0.001	2.01 (1.47-2.74)	< 0.001	1.88 (1.31-2.70)	0.001
**Alcohol intake** (≥10 g/day *vs*. <10g/day)			2.08 (1.12-3.87)	0.021	2.24 (1.18-4.28)	0.014	3.44 (1.61-7.36)	0.001
**Current smoking** (yes/no)			0.94 (0.75-1.18)	0.593	0.89 (0.71-1.12)	0.318	0.99 (0.76-1.30)	0.956
**eGFR** (mL/min/1.73 m^2)					0.98 (0.97-0.98)	<0.001	0.97 (0.97-0.98)	< 0.001
**UAE** (mg/24-h)					1.00 (1.00-1.00)	0.562	1.00 (1.00-1.00)	0.192
**Use of antihypertensive medication** (yes/no)					1.28 (0.92-1.77)	0.144	0.91 (0.62-1.33)	0.616
**Use of glucose lowering drugs** (yes/no)					0.97 (0.46-2.04)	0.928	1.42 (0.60-3.37)	0.428
**Use of lipid lowering drugs** (yes/no)					1.42 (1.01-2.00)	0.043	1.15 (0.77-1.71)	0.505
**HOMA-IR** (mU mmol/L^2/22.5)							13.00 (10.10-16.73)	< 0.001
**Men**								
	OR (95%CI)	*P* value	OR (95%CI)	*P* value	OR (95%CI)	*P* value	OR (95%CI)	*P* value
**Age** (years)	1.03 (1.02-1.03)	< 0.001	1.00 (1.00-1.01)	0.413	0.99 (0.98-1.00)	0.076	0.99 (0.98-1.00)	0.118
**Sodium excretion per 24-h** (1 SD increment)	1.48 (1.37-1.59)	< 0.001	1.46 (1.35-1.57)	< 0.001	1.48 (1.36-1.60)	< 0.001	1.24 (1.13-1.36)	< 0.001
**Type 2 diabetes mellitus** (yes/no)			2.06 (1.51-2.81)	< 0.001	2.12 (1.29-3.49)	0.003	0.36 (0.20-0.65)	0.001
**History of cardiovascular disease** (yes/no)			0.90 (0.69-1.19)	0.465	0.72 (0.52-1.00)	0.050	0.69 (0.48-1.00)	0.048
**Hypertension** (yes/no)			3.08 (2.57-3.69)	< 0.001	2.54 (2.01-3.22)	< 0.001	2.12 (1.63-2.77)	< 0.001
**Alcohol intake** (≥10 g/day *vs*. <10g/day)			1.43 (1.05-1.95)	0.023	1.52 (1.10-2.09)	0.011	1.87 (1.29-2.71)	0.001
**Current smoking** (yes/no)			1.20 (1.01-1.44)	0.038	1.19 (0.99-1.42)	0.067	1.44 (1.17-1.78)	0.001
**eGFR** (mL/min/1.73 m^2)					0.99 (0.99-1.00)	0.007	1.00 (0.99-1.01)	0.762
**UAE** (mg/24-h)					1.00 (1.00-1.00)	0.003	1.00 (1.00-1.00)	0.005
**Use of antihypertensive medication** (yes/no)					1.22 (0.93-1.61)	0.149	1.12 (0.82-1.54)	0.470
**Use of glucose lowering drugs** (yes/no)					0.90 (0.48-1.70)	0.752	2.25 (1.09-4.66)	0.029
**Use of lipid lowering drugs** (yes/no)					1.47 (1.10-1.97)	0.010	1.27 (0.92-1.76)	0.147
**HOMA-IR** (mU mmol/L^2/22.5)							7.71 (6.33-9.38)	< 0.001

OR, odds ratio; 95%CI, 95% confidence intervals. OR is given per 1 SD increase for sodium excretion. 1 SD change in urinary sodium excretion corresponds to 55.99 mmol sodium per day. HOMA-IR was $\log_e$ transformed for analyses. Abbreviations: 24-h, twenty-four hours; eGFR, estimated glomerular filtration rate; FLI, Fatty Liver Index; HOMA-IR, Homeostasis Model Assessment of Insulin Resistance; UAE; urinary albumin excretion.

Model 1: adjusted for age.

Model 2: adjusted for age, presence of type 2 diabetes, history of cardiovascular disease, presence of hypertension, alcohol intake and current smoking.

Model 3: adjusted for age, presence of type 2 diabetes, history of cardiovascular disease, presence of hypertension, alcohol intake, current smoking, estimated glomerular filtration rate, urinary albumin excretion, use of antihypertensive medication, glucose lowering drugs and lipid lowering drugs.

Model 4: adjusted for age, presence of type 2 diabetes, history of cardiovascular disease, presence of hypertension, alcohol intake, current smoking, estimated glomerular filtration rate, urinary albumin excretion, use of antihypertensive medication, glucose lowering drugs and lipid lowering drugs and HOMA-IR.

Table S2. Multivariable regression analysis demonstrating the positive association of an elevated Hepatic Steatosis Index (HSI > 36) with averaged 24-h sodium excretion (two collections) after adjustment for clinical and laboratory covariates in 3,100 women and in 3,032 men separately.

	**Model 1**		**Model 2**		**Model 3**		**Model 4**	
**Women**								
	OR (95%CI)	*P* value	OR (95%CI)	*P* value	OR (95%CI)	*P* value	OR (95%CI)	*P* value
**Age** (years)	1.05 (1.04-1.06)	< 0.001	1.03 (1.02-1.03)	< 0.001	1.01 (1.00-1.02)	0.012	1.01 (1.00-1.02)	0.138
**Sodium excretion per 24-h** (1 SD increment)	1.68 (1.52-1.85)	< 0.001	1.65 (1.48-1.83)	< 0.001	1.66 (1.49-1.85)	< 0.001	1.51 (1.34-1.69)	< 0.001
**Type 2 diabetes mellitus** (yes/no)			7.46 (4.93-11.27)	< 0.001	8.03 (4.25-15.16)	< 0.001	3.05 (1.51-6.14)	0.002
**History of cardiovascular disease** (yes/no)			1.22 (0.78-1.90)	0.392	0.94 (0.57-1.53)	0.791	0.81 (0.48-1.37)	0.426
**Hypertension** (yes/no)			2.27 (1.86-2.77)	< 0.001	1.85 (1.39-2.47)	< 0.001	1.78 (1.32-2.41)	< 0.001
**Alcohol intake** (≥10 g/day *vs*. <10g/day)			1.04 (0.54-1.98)	0.918	1.02 (0.52-2.02)	0.951	1.11 (0.54-2.28)	0.771
**Current smoking** (yes/no)			0.63 (0.52-0.77)	< 0.001	0.60 (0.49-0.74)	<0.001	0.61 (0.49-0.77)	< 0.001
**eGFR** (mL/min/1.73 m^2)					0.99 (0.98-1.00)	0.002	0.99 (0.98-1.00)	0.021
**UAE** (mg/24-h)					1.00 (1.00-1.00)	0.791	1.00 (1.00-1.00)	0.750
**Use of antihypertensive medication** (yes/no)					1.28 (0.94-1.74)	0.124	1.04 (0.75-1.44)	0.821
**Use of glucose lowering drugs** (yes/no)					0.91 (0.39-2.11)	0.821	0.96 (0.39-2.35)	0.933
**Use of lipid lowering drugs** (yes/no)					1.32 (0.95-1.83)	0.102	1.14 (0.80-1.61)	0.463
**HOMA-IR** (mU mmol/L^2/22.5)							3.17 (2.67-3.77)	< 0.001
**Men**								
	OR (95%CI)	*P* value	OR (95%CI)	*P* value	OR (95%CI)	*P* value	OR (95%CI)	*P* value
**Age** (years)	1.00 (1.00-1.01)	0.598	0.98 (0.97-0.99)	< 0.001	0.97 (0.96-0.98)	< 0.001	0.97 (0.96-0.98)	< 0.001
**Sodium excretion per 24-h** (1 SD increment)	1.62 (1.50-1.75)	< 0.001	1.57 (1.45-1.70)	< 0.001	1.56 (1.44-1.70)	< 0.001	1.34 (1.23-1.47)	< 0.001
**Type 2 diabetes mellitus** (yes/no)			4.33 (3.18-5.89)	< 0.001	4.35 (2.69-7.05)	< 0.001	1.18 (0.69-2.03)	0.548
**History of cardiovascular disease** (yes/no)			0.84 (0.62-1.15)	0.274	0.72 (0.50-1.03)	0.068	0.69 (0.47-1.03)	0.067
**Hypertension** (yes/no)			2.49 (2.04-3.05)	< 0.001	2.12 (1.64-2.74)	< 0.001	1.64 (1.24-2.17)	< 0.001
**Alcohol intake** (≥10 g/day *vs*. <10g/day)			1.35 (0.97-1.90)	0.079	1.44 (1.02-2.04)	0.037	1.57 (1.07-2.29)	0.021
**Current smoking** (yes/no)			0.84 (0.69-1.02)	0.075	0.83 (0.68-1.02)	0.079	0.91 (0.73-1.13)	0.390
**eGFR** (mL/min/1.73 m^2)					0.99 (0.99-1.00)	0.120	1.00 (1.00-1.01)	0.494
**UAE** (mg/24-h)					1.00 (1.00-1.00)	0.285	1.00 (1.00-1.00)	0.512
**Use of antihypertensive medication** (yes/no)					1.20 (0.90-1.61)	0.215	1.05 (0.76-1.44)	0.783
**Use of glucose lowering drugs** (yes/no)					0.88 (0.48-1.60)	0.669	1.82 (0.94-3.56)	0.078
**Use of lipid lowering drugs** (yes/no)					1.37 (1.00-1.87)	0.046	1.23 (0.88-1.72)	0.225
**HOMA-IR** (mU mmol/L^2/22.5)							5.10 (4.23-6.15)	< 0.001

OR, odds ratio; 95%CI, 95% confidence intervals. OR is given per 1 SD increase for sodium excretion. 1 SD change in urinary sodium excretion corresponds to 55.99 mmol sodium per day. HOMA-IR was $\log_e$ transformed for analyses. Abbreviations: 24-h, twenty-four hours; eGFR, estimated glomerular filtration rate; HOMA-IR, Homeostasis Model Assessment of Insulin Resistance; HSI, Hepatic Steatosis Index; UAE; urinary albumin excretion.

Model 1: adjusted for age.

Model 2: adjusted for age, presence of type 2 diabetes, history of cardiovascular disease, presence of hypertension, alcohol intake and current smoking.

Model 3: adjusted for age, presence of type 2 diabetes, history of cardiovascular disease, presence of hypertension, alcohol intake, current smoking, estimated glomerular filtration rate, urinary albumin excretion, use of antihypertensive medication, glucose lowering drugs and lipid lowering drugs.

Model 4: adjusted for age, presence of type 2 diabetes, history of cardiovascular disease, presence of hypertension, alcohol intake, current smoking, estimated glomerular filtration rate, urinary albumin excretion, use of antihypertensive medication, glucose lowering drugs and lipid lowering drugs and HOMA-IR.

Table S3. Sensitivity analyses demonstrating the positive association of an elevated Fatty Liver Index (FLI ≥ 60) with 24-h sodium excretion in 3,221 subjects after excluding subjects with alcohol intake ≥10 g/day, a positive cardiovascular history, presence of hypertension, impaired estimated glomerular filtration rate (<60 mL/min/1.73 m^2), elevated urinary albumin excretion (>30 mg/24 hr), use of antihypertensive drugs, glucose lowering drugs and lipid lowering drugs.

	**Model 1**		**Model 2**		**Model 3**	
	OR (95%CI)	*P* value	OR (95%CI)	*P* value	OR (95%CI)	*P* value
**Age** (years)	1.02 (1.02-1.03)	< 0.001	1.02 (1.01-1.03)	< 0.001	1.01 (1.00-1.02)	0.053
**Sex** (men *vs.* women)	2.49 (2.05-3.03)	< 0.001	2.49 (2.05-3.03)	< 0.001	2.82 (2.25-3.54)	< 0.001
**Sodium excretion per 24-h** (1 SD increment)	1.43 (1.30-1.58)	< 0.001	1.44 (1.31-1.59)	< 0.001	1.27 (1.13-1.42)	< 0.001
**Type 2 diabetes mellitus** (yes/no)			3.57 (1.87-6.81)	< 0.001	0.38 (0.17-0.83)	0.016
**Current smoking** (yes/no)			1.14 (0.93-1.40)	0.208	1.31 (1.04-1.66)	0.025
**HOMA-IR** (mU mmol/L^2/22.5)					11.79 (9.33-14.90)	< 0.001

OR, odds ratio; 95%CI, 95% confidence intervals. OR is given per 1 SD increase for sodium excretion. 1 SD change in urinary sodium excretion corresponds to 55.99 mmol sodium per day. HOMA-IR was $\log_e$ transformed for analyses. Abbreviations: 24-h, twenty-four hours; eGFR, estimated glomerular filtration rate; FLI, Fatty Liver Index; HOMA-IR, Homeostasis Model Assessment of Insulin Resistance; UAE; urinary albumin excretion.

Model 1: adjusted for age and sex.

Model 2: adjusted for age, sex, presence of type 2 diabetes and current smoking.

Model 3: adjusted for age, sex, presence of type 2 diabetes, current smoking and HOMA-IR.

Table S4. Sensitivity analyses demonstrating the positive association of an elevated Hepatic Steatosis Index (HSI > 36) with 24-h sodium excretion in 3,221 subjects after excluding subjects with alcohol intake ≥10 g/day, a positive cardiovascular history, presence of hypertension, impaired estimated glomerular filtration rate (<60 mL/min/1.73 m^2), elevated urinary albumin excretion (>30 mg/24 hr), use of antihypertensive drugs, glucose lowering drugs and lipid lowering drugs.

	**Model 1**		**Model 2**		**Model 3**	
	OR (95%CI)	*P* value	OR (95%CI)	*P* value	OR (95%CI)	*P* value
**Age** (years)	1.01 (1.00-1.02)	0.005	1.01 (1.00-1.02)	0.024	1.00 (0.99-1.01)	0.625
**Sex** (men *vs*. women)	0.80 (0.66-0.96)	0.016	0.79 (0.65-0.95)	0.014	0.69 (0.56-0.85)	< 0.001
**Sodium excretion per 24-h** (1 SD increment)	1.47 (1.34-1.62)	< 0.001	1.48 (1.35-1.63)	< 0.001	1.35 (1.22-1.50)	< 0.001
**Type 2 diabetes mellitus** (yes/no)			4.76 (2.55-8.87)	< 0.001	1.00 (0.48-2.09)	0.994
**Current smoking** (yes/no)			0.74 (0.61-0.91)	0.005	0.76 (0.61-0.95)	0.016
**HOMA-IR** (mU mmol/L^2/22.5)					6.04 (4.97-7.32)	< 0.001

OR, odds ratio; 95%CI, 95% confidence intervals. OR is given per 1 SD increase for sodium excretion. 1 SD change in urinary sodium excretion corresponds to 55.99 mmol sodium per day. HOMA-IR was $\log_e$ transformed for analyses. Abbreviations: 24-h, twenty-four hours; eGFR, estimated glomerular filtration rate; HOMA-IR, Homeostasis Model Assessment of Insulin Resistance; HSI, Hepatic Steatosis Index; UAE; urinary albumin excretion.

Model 1: adjusted for age and sex.

Model 2: adjusted for age, sex, presence of type 2 diabetes and current smoking.

Model 3: adjusted for age, sex, presence of type 2 diabetes, current smoking and HOMA-IR.
